# Retraction: Novel hybrid QSPR-GPR approach for modeling of carbon dioxide capture using deep eutectic solvents

**DOI:** 10.1039/d4ra90073a

**Published:** 2024-06-27

**Authors:** Iman Salahshoori, Alireza Baghban, Amirhosein Yazdanbakhsh

**Affiliations:** a Department of Chemical Engineering, Science and Research Branch, Islamic Azad University Tehran Iran; b Department of Process Engineering, NISOC Company Ahvaz Iran Alireza_baghban@ut.ac.ir; c Research and Development Manager, Teb Plastic Company Tehran Iran

## Abstract

Retraction of ‘Novel hybrid QSPR-GPR approach for modeling of carbon dioxide capture using deep eutectic solvents’ by Iman Salahshoori *et al.*, *RSC Adv.*, 2023, **13**, 30071–30085, https://doi.org/10.1039/d3ra05360a.

The Royal Society of Chemistry hereby wholly retracts this *RSC Advances* article due to unattributed text and data overlap with a *Green Chemistry* article by different authors.^1^

This *Green Chemistry* article was cited as ref. 24 in the paper, however it was not made clear that the methods and the data in the supplementary information were taken from the previous publication.

When the authors were asked to provide the output files from the optimisations, the files provided were generated after publication and were not optimisations, instead were simple single point calculations of the energy, with the structures made by hand.

In addition, the Royal Society of Chemistry has been contacted by the University of KwaZulu-Natal and Iran Polymer and Petrochemical Institute to inform the editor that the affiliations for Iman Salahshoori were listed incorrectly in the original manuscript. They were listed due to the author's involvement with multiple institutions at the time of submission, however this was an oversight, and the work contained in this article was not associated with the other institutions. The corrected list of affiliations for this paper is as shown here.

The authors were informed about the retraction of the article. Iman Salahshoori and Alireza Baghban have not agreed with the decision, Amirhosein Yazdanbakhsh has not responded.

Signed: Laura Fisher, Executive Editor, *RSC Advances*

Date: 21st June 2024
